# New guidelines on primary PCI for patients with STEMI: changing insights

**DOI:** 10.1007/s12471-015-0780-8

**Published:** 2015-12-08

**Authors:** E.E. van der Wall

**Affiliations:** Netherlands Society of Cardiology/Holland Heart House, Moreelsepark 1, 3511 EP Utrecht, The Netherlands

Recently, the American College of Cardiology, American Heart Association, and Society for Cardiovascular Angiography and Interventions (ACC/AHA/SCAI) have updated their guidelines on primary percutaneous coronary intervention (PCI) for patients with ST-segment-elevation myocardial infarction (STEMI). These organisations have made several remarkable changes from previous recommendations. The new guidelines were published in the Journal of the American College of Cardiology in October 2015 [1].

The background for the 2015 focused update was novel findings from clinical trials presented at the major cardiology congresses from 2013 to 2015 and new information from other peer-reviewed studies published up to August 2015. These recent studies were thoroughly reviewed by the 2011 PCI and 2013 STEMI Guideline Writing Committees and the Task Force to identify trials and other crucial data that might affect guideline recommendations. This information was considered important enough to prompt updated recommendations and was included in evidence tables in the Online Data Supplement to be found on:

http://jaccjacc.acc.org/Clinical_Document/2015_Focused_Update_on_Primary_PCI_in_STEMI_Data_Supplements.pdf)

The Classes of Recommendation and Levels of Evidence were derived independently of one another according to established criteria. Five different classes were distinguished: Class I (Strong), IIa (Moderate), IIb (Weak), III (No benefit, moderate), and Class III (Harm, strong). Also, five levels of evidence were used: Level A, B-R (randomised), B-NR (non-randomised), C-LD (limited data), and Level C-EO (expert opinion).

The scope of the 2015 focused update was restricted to two main areas: 1) multi-vessel PCI, and 2) thrombus aspiration in patients with STEMI undergoing primary PCI.

1. *Culprit artery-only PCI versus multi-vessel PCI*

The 2013 recommendation was Class III (Harm), indicating that PCI should not be performed in a non-infarct artery at the time of primary PCI in patients with STEMI who are haemodynamically stable (Level of Evidence B).

The new recommendation of 2015 is now Class IIb, demonstrating that PCI of a non-infarct artery may be considered in selected patients with STEMI and multi-vessel disease who are haemodynamically stable, either at the time of primary PCI or as a planned staged procedure (Level of Evidence B-R). It was commented that the 2015 recommendation changed from Class III (Harm) to Class IIb and expanded the time frame in which multi-vessel PCI could be performed.

2) *Aspiration thrombectomy*

Recommendations of 2011/2013 were Class IIa, indicating that manual aspiration thrombectomy is reasonable for patients undergoing primary PCI (Level of Evidence B).

The new recommendation of 2015 is Class IIb, showing that the usefulness of selective and bailout aspiration thrombectomy in patients undergoing primary PCI is not well established (Level of Evidence C-LD). The recommendation of 2015 for routine aspiration thrombectomy is now Class III (No benefit), indicating that routine aspiration thrombectomy before primary PCI is not useful (Level of Evidence A).

It was commented that the 2015 recommendation changed from Class IIa to Class IIb for selective and bailout aspiration thrombectomy before PCI and that routine aspiration thrombectomy before primary PCI is not useful.

In summary, the 2015 report has significantly updated the 2011 ACC/AHA/SCAI Guideline for PCI and the 2013 ACC/AHA Guideline for the Management of STEMI. The 2015 focused update did address two major issues: 1) the setting of primary PCI for multi-vessel PCI, and 2) the usefulness of thrombus aspiration. Regarding multi-vessel PCI, new evidence from recent clinical trials clearly showed that treating other non-culprit stenosed coronary arteries may be safe and beneficial in selected patients with multi-vessel disease. The 2015 focused update states that treating the non-culprit stenosed arteries with a stent may be considered in patients with STEMI who are haemodynamically stable at the time of the primary PCI. Concerning the usefulness of thrombus aspiration, the prior Class IIa recommendation for aspiration thrombectomy was changed to Class IIb in 2015. There are at present insufficient data to assess the potential benefit of a strategy of selective or bailout aspiration thrombectomy. The 2015 focused update states that routine aspiration thrombectomy before primary PCI is currently not recommended.

The above-mentioned new recommendations may also have consequences for newly planned guidelines of the European Society of Cardiology (ESC) on STEMI. In 2012 the ESC STEMI guidelines reported: 1) that primary PCI should be limited to the culprit vessel with the exception of cardiogenic shock and persistent ischaemia after PCI of the supposed culprit lesion (Class IIa, Level B), and 2) that routine thrombus aspiration should be considered (Class IIa, Level B) [2]. The updated ESC STEMI guidelines are among the new publications scheduled in 2017.

What can be learned from new recommendations and guidelines? Generally speaking, according to the definition of the ESC, guidelines aim to present all the relevant evidence on a particular clinical issue in order to help physicians to weigh the benefits and risks of a particular diagnostic or therapeutic procedure [3–6]. They should be helpful in everyday clinical medical decision-making. More importantly, guidelines should be updated every 4 to 5 years in order to offer the patient the best possible treatment based on the most actual findings [7–11]. An updated guideline, however, does not necessarily invalidate the previous guideline; a new guideline predominantly reflects the progress in science and its ensuing change of views over time. New guidelines are built on older guidelines based on moving insights inspired by advanced research [12–14]. In itself, each guideline represents the level of science at that particular point in time. Fortunately, progress in research teaches us that the level of science moves forward implying that guidelines have to be regularly revisited, primarily in the interest of optimal diagnosis and treatment of the patient [15–18]. In this respect, the 2015 focused update on primary PCI for patients with STEMI offers an illustrative example of changing insights based on novel findings [1].
